# A Lung Ultrasound Radiomics-Based Machine Learning Model for Diagnosing Acute Heart Failure in the Emergency Department

**DOI:** 10.3390/diagnostics16040598

**Published:** 2026-02-17

**Authors:** Jifei Cai, Nan Tong, Chenchen Hang, Xuan Qi, Lulu Su, Shubin Guo

**Affiliations:** Emergency Medicine Clinical Research Center, Beijing Chao-Yang Hospital, Capital Medical University, Beijing 100020, China; tongnan1105@126.com (N.T.); hchch0926@126.com (C.H.); cynthiatry@163.com (X.Q.); sddpsululu@163.com (L.S.)

**Keywords:** acute heart failure, lung ultrasound, radiomics, machine learning, random forest, emergency department, diagnostic model, multimodal fusion

## Abstract

**Background/Objectives**: Acute heart failure (AHF) is a common critical condition in emergency departments, and traditional diagnostic methods have limitations, including high subjectivity and limited accuracy. This study aimed to develop an integrated machine learning model based on lung ultrasound (LUS) radiomics and clinical data for diagnosing AHF in patients presenting with acute dyspnea. **Methods**: A total of 301 patients were included and randomly split into training (*n* = 210) and testing (*n* = 91) sets. Using PyRadiomics 3.0, 107 radiomics features were extracted from standardized 6-zone LUS images, combined with 52 clinical features. Three random forest models were developed: clinical-only, radiomics-only, and integrated models. **Results**: The integrated model achieved optimal performance on the testing set with an AUC of 0.976 (95% CI: 0.950–0.994), accuracy of 90.1%, sensitivity of 91.1%, and specificity of 89.1%, significantly outperforming the radiomics model (AUC 0.940, *p* = 0.046) and clinical model (AUC 0.931, *p* = 0.111). Feature importance analysis revealed that radiomics features contributed 75.6% of the model’s predictive power, with gray level run length matrix (GLRLM) features dominating the top-ranked features. **Conclusions**: As a proof-of-concept study, this research demonstrates the potential value of multimodal data fusion strategies for AHF diagnosis in the emergency department; however, external validation and prospective studies are required to further confirm its clinical applicability.

## 1. Introduction

Acute heart failure (AHF) is one of the major causes of hospitalization worldwide, imposing a heavy burden on healthcare systems. In the United States, approximately 1 million patients are hospitalized annually for AHF, with in-hospital mortality rates of 4–7% and 30-day readmission rates up to 25%, severely affecting patient prognosis and quality of life [1,2]. The emergency department (ED) is the primary site for initial presentation of AHF patients, with approximately 80% of AHF patients admitted through the ED [3]. However, early accurate diagnosis of AHF faces numerous challenges due to highly heterogeneous clinical presentations that often overlap with other causes of dyspnea, such as chronic obstructive pulmonary disease, pneumonia, and pulmonary embolism [4]. Studies show that emergency physicians’ initial diagnostic accuracy for AHF is only about 80%, with significant risks of misdiagnosis and missed diagnosis [5].

Traditional diagnostic methods rely on history taking, physical examination, chest X-ray, and biomarkers such as NT-proBNP, but all these methods have limitations. Physical examination has low sensitivity and specificity; chest X-ray diagnostic accuracy for pulmonary edema is limited by image quality and interpreter experience; although NT-proBNP is widely used, its diagnostic value is affected by multiple factors including age, renal function, obesity, and atrial fibrillation, with gray zones and uncertainties [6]. Therefore, there is an urgent need to develop new, more accurate AHF diagnostic tools to improve clinical decision-making and patient management in the ED.

In recent years, lung ultrasound (LUS) has shown great potential in AHF diagnosis as a bedside, non-invasive, real-time examination method. LUS assesses alveolar interstitial syndrome and extravascular lung water by detecting B-lines—laser-like hyperechoic vertical artifacts originating from the pleural line [7,8]. Multiple studies have confirmed that LUS is superior to traditional physical examination and chest X-ray in diagnosing pulmonary edema. A 2024 study showed that applying LUS in prehospital emergency settings significantly improved diagnostic sensitivity and accuracy of AHF [9]. The 2023 expert consensus released by the European Association of Cardiovascular Imaging further clarified the clinical application value of LUS in acute and chronic heart failure [10]. However, traditional LUS assessment mainly relies on operator subjective judgment and manual B-line counting, which has problems such as high inter-operator variability and difficulty in standardization, limiting its widespread clinical application.

Radiomics provides an innovative approach to solving these problems. Radiomics refers to high-throughput extraction of quantitative features from medical images and analysis through statistical and machine learning methods to reveal imaging phenotypes related to disease diagnosis, prognosis, and treatment response [11,12]. Unlike traditional subjective image interpretation, radiomics can extract high-order texture features that are difficult for the human eye to identify, objectively quantifying spatial heterogeneity and microstructural characteristics of tissues [13]. In LUS applications, radiomics can precisely quantify texture patterns such as B-line density, distribution, continuity, and directionality, overcoming the limitations of subjective assessment [14]. Machine learning algorithms, particularly random forest, can integrate high-dimensional radiomics features with clinical data to build powerful diagnostic models [15]. The combination of radiomics and machine learning has made significant progress in tumor diagnosis and prognosis assessment, demonstrating great application potential in precision medicine [16,17].

The pathophysiological mechanisms of AHF are complex, involving interactions among cardiac dysfunction, hemodynamic disturbances, neuroendocrine activation, and renal impairment. Single data modalities cannot comprehensively reflect the disease complexity. Multimodal data fusion, integrating radiomics features with clinical data, can capture disease information from multiple dimensions, providing more comprehensive diagnostic evidence [18]. A 2024 study showed that a multimodal radiomics model integrating ultrasound, mammography, and MRI achieved an AUC of 0.942 in distinguishing benign from malignant breast tumors, significantly outperforming single-modality models [19]. However, current research on integrating LUS radiomics with clinical data for AHF diagnosis remains relatively scarce.

Although previous studies have explored the application of machine learning in heart failure diagnosis, the present study offers several novel contributions. First, this study is the first to systematically apply 107 IBSI-compliant radiomics features to LUS image analysis, enabling quantitative characterization of B-line patterns and overcoming the limitations of traditional semi-quantitative scoring methods. Second, the model performance was validated in a real-world emergency department population without excluding patients with common comorbidities such as COPD and chronic kidney disease, thereby enhancing the external validity of the findings. Third, through feature importance analysis, this study revealed the central role of GLRLM texture features in AHF diagnosis, providing novel insights into the biological basis of LUS radiomics. Fourth, this study evaluated the relative contributions of clinical and radiomics features (approximately 1:3), quantifying the incremental value of multimodal fusion [20,21].

Based on the above background and research gaps, this study aimed to develop and validate a machine learning-based integrated model of LUS radiomics and clinical data for diagnosing AHF in ED patients with acute dyspnea. Specific objectives included: (1) extracting high-dimensional radiomics features from standardized 6-zone LUS images using the PyRadiomics platform; (2) integrating radiomics features with 52 clinical parameters to build three random forest diagnostic models; (3) evaluating discrimination and calibration abilities of each model on an independent testing set; (4) applying feature importance analysis to identify key diagnostic factors and enhance model interpretability; and (5) to validate model performance in a real-world emergency department population including patients with comorbidities, as well as to evaluate preliminary evidence as a proof-of-concept study.

## 2. Materials and Methods

### 2.1. Study Design and Ethical Approval

This was a single-center retrospective diagnostic accuracy study conducted in the emergency department of Beijing Chaoyang Hospital, Capital Medical University. Patients presenting to the ED with acute dyspnea between October 2024 and October 2025 were included. The study was approved by the Ethics Committee of Beijing Chaoyang Hospital, Capital Medical University (Approval No.: 2025-6-5-1), and informed consent was waived due to the retrospective nature of the study. The study followed the principles of the Declaration of Helsinki. All data were retrieved from the hospital’s electronic medical record (EMR) system and picture archiving and communication system (PACS) and were fully de-identified to protect patient privacy. Reporting followed the TRIPOD and STARD guidelines.

### 2.2. Study Population

Inclusion criteria were: (1) age ≥ 18 years; (2) presenting to the ED with acute dyspnea, defined as acute onset within 48 h, respiratory rate > 20 breaths/min, and peripheral oxygen saturation < 90% on room air; (3) completed standardized 6-zone LUS examination with adequate image quality for radiomics analysis; (4) completed echocardiography with measurement of left ventricular ejection fraction (LVEF) and cardiac chamber dimensions; (5) completed laboratory tests including NT-proBNP, cardiac biomarkers, and renal function; and (6) complete clinical data allowing definitive AHF diagnosis determination by the expert team.

**Figure 1:**
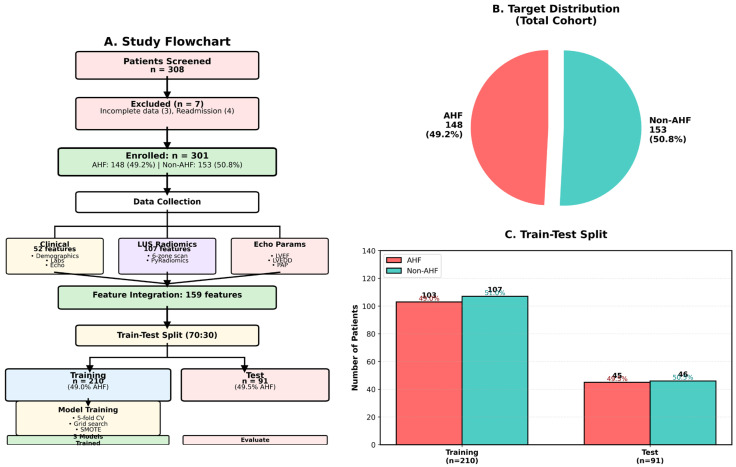
Patient selection flowchart, class distribution, and stratified dataset partitioning. (**A**) Study flowchart showing patient screening, exclusion, data collection, feature integration, and train–test split. (**B**) Pie chart of overall cohort class distribution. (**C**) Bar chart of AHF and non-AHF patient counts in training and testing sets. In panels (**B**,**C**), coral represents the AHF group and teal represents the non-AHF group.

Exclusion criteria were: (1) poor LUS image quality preventing radiomics feature extraction; (2) missing key clinical data including NT-proBNP, LVEF, and serum creatinine; (3) multiple admissions during the study period (only first admission included); (4) uncertain final diagnosis; and (5) other life-threatening emergencies requiring immediate intervention. This study did not exclude patients with comorbidities such as chronic obstructive pulmonary disease, asthma, or chronic kidney disease to ensure clinical representativeness. A total of 308 patients were screened, with 7 excluded (3 with incomplete data, 4 repeat admissions), resulting in 301 patients in the final analysis (Figure 1A).

### 2.3. Reference Standard for Diagnosis

AHF diagnosis used a comprehensive clinical assessment as the reference standard, independently determined by two senior attending cardiologists (both with > 10 years of AHF diagnostic experience), blinded to LUS radiomics features but with access to the original LUS images. Diagnostic criteria included: (1) comprehensive clinical assessment with detailed history and physical examination; (2) transthoracic echocardiography assessment of LVEF, cardiac chamber dimensions, and valvular function; (3) laboratory tests including NT-proBNP, troponin I, and renal function; and (4) other ancillary tests when necessary. Diagnosis was based on the 2021 European Society of Cardiology guidelines and Framingham heart failure diagnostic criteria. The two experts independently evaluated all clinical data; in case of disagreement, a third senior attending emergency physician adjudicated. Final diagnosis required consensus by at least 2 experts.

It should be noted that, as part of the reference standard, expert adjudicators had access to the original LUS images (including B-line assessment) but were completely blinded to the radiomics feature analysis results. Since B-lines constitute a component of the AHF diagnostic criteria [3], this design may introduce potential incorporation bias, as the reference standard utilized the same imaging modality as the model being tested. This limitation may artificially inflate the apparent diagnostic performance of the model and should be considered when interpreting the results.

### 2.4. Clinical Data Collection

Complete clinical data were extracted from the EMR system, totaling 52 features, including: demographic information (age, sex, BMI); vital signs (systolic and diastolic blood pressure, heart rate, respiratory rate, peripheral oxygen saturation); laboratory tests (30 items including NT-proBNP, troponin I, CK-MB, creatinine, BUN, eGFR, electrolytes, glucose, complete blood count, CRP, and lipid profile); echocardiographic parameters (LVEF, LVEDD, LVESD, PAP); and comorbidities (hypertension, diabetes mellitus, coronary artery disease, chronic heart failure history, atrial fibrillation, cerebrovascular disease, COPD, chronic kidney disease, peripheral vascular disease, malignancy).

### 2.5. Lung Ultrasound Examination

All patients underwent LUS examination using the Mindray M9 ultrasound system (Mindray Bio-Medical Electronics Co., Ltd., Shenzhen, China). A convex probe (Model: C5-2, frequency 2–5 MHz) was used with depth set at 10 cm. Examinations were performed by 2 emergency department attending physicians who received professional training (completing > 100 LUS scans). The two operators underwent consistency testing with a Kappa coefficient > 0.85.

A simplified 6-zone scanning protocol was used, dividing each hemithorax into 3 zones: right anterior zone (R1, mid-clavicular line, 2nd–4th intercostal spaces), right lateral zone (R2, mid-axillary line, 3rd–5th intercostal spaces), right posterior zone (R3, subscapular line, 7th–9th intercostal spaces), and corresponding left zones (L1, L2, L3). Patients were positioned semi-recumbent or supine. Three static images were acquired at end-inspiration for each zone, with the operator selecting 1 image with optimal quality for storage. All images were stored in DICOM format in the PACS.

### 2.6. Radiomics Feature Extraction

All LUS images in DICOM format were imported into 3D Slicer software (version 5.6.2) for preprocessing, including image format conversion, gray-level normalization, and denoising. Region of interest (ROI) delineation was performed by an experienced radiologist (>5 years of ultrasound image analysis experience), encompassing the pleural line and lung parenchyma below it. To assess ROI delineation reliability, images from 30 randomly selected patients were independently delineated by another radiologist, and the intraclass correlation coefficient (ICC) was calculated. ICC > 0.90 indicated good reproducibility.

A total of 107 standardized radiomics features were extracted using the PyRadiomics 3.0 platform [22], which follows the Image Biomarker Standardization Initiative (IBSI) guidelines [23]. Extracted features included: shape features (14), first-order statistics features (18), and texture features (75, including gray level co-occurrence matrix [GLCM, 24], gray level run length matrix [GLRLM, 16], gray level size zone matrix [GLSZM, 16], gray level dependence matrix [GLDM, 14], and neighborhood gray tone difference matrix [NGTDM, 5]).

### 2.7. Dataset Partitioning and Preprocessing

The 301 patients were randomly split into a training set (*n* = 210, 70%) and a testing set (*n* = 91, 30%) at a 7:3 ratio using stratified random sampling to ensure AHF prevalence consistency. The train_test_split function from Python’s scikit-learn library was used with parameters test_size = 0.3, random_state = 42, stratify = AHF labels. Overall cohort AHF prevalence was 49.2% (148/301), training set 49.0% (103/210), and testing set 49.5% (45/91), all highly consistent (*p* > 0.99, Chi-square test) (Figure 1C).

To address mild class imbalance in the training set, the Synthetic Minority Over-sampling Technique (SMOTE) [24] was applied. After resampling, the training set achieved a perfect 1:1 balance (AHF 107 cases vs. non-AHF 107 cases, total 214 cases). Feature standardization used StandardScaler for Z-score standardization of all features.

Regarding sample size, this study included 301 patients and 159 features, yielding a feature-to-event ratio of approximately 1:0.93 (148 positive events/159 features). Although this ratio falls below the traditional 10:1 standard recommended for conventional statistical models, ensemble learning methods such as random forest demonstrate better tolerance to high-dimensional feature spaces [25]. Furthermore, we employed 5-fold cross-validation and independent test set validation to assess model generalizability and mitigate overfitting risk. It should be acknowledged that SMOTE resampling may introduce optimistic bias, particularly in small sample settings [26]. In this study, SMOTE was applied exclusively to the training set, while the test set retained its original distribution to ensure unbiased performance evaluation.

### 2.8. Machine Learning Model Development

Three complementary random forest diagnostic models were developed: (1) clinical-only model, trained using only 52 clinical features; (2) radiomics-only model, trained using only 107 LUS radiomics features; and (3) integrated model, fusing all 159 features. All models employed a Random Forest classifier implemented using Python 3.11.10 and scikit-learn 1.3.0 library.

Hyperparameters were optimized through grid search combined with 5-fold stratified cross-validation. The search space included: n_estimators (100, 200, 300), max_depth (10, 20, 30), min_samples_split (2, 5, 10), and min_samples_leaf (1, 2). Area under the receiver operating characteristic curve (AUC) was used as the optimization objective.

### 2.9. Model Evaluation

Discrimination ability was evaluated on the independent testing set, calculating AUC and 95% confidence interval (CI) using bootstrap resampling (n = 1000), sensitivity, specificity, accuracy, positive predictive value (PPV), negative predictive value (NPV), and F1-score. The DeLong test was used to compare AUC differences between models. Calibration performance was assessed using calibration curves and quantitative calibration metrics, including Brier score, calibration slope, calibration intercept, and expected calibration error (ECE). Feature importance was calculated using the mean decrease in impurity (MDI) method.

To comprehensively evaluate model calibration performance, the following quantitative metrics were calculated [27]: (1) Brier score, which measures the overall accuracy of probability predictions, ranging from 0 to 1, with lower values indicating better performance; (2) calibration slope, with an ideal value of 1.0, where values < 1.0 indicate overconfident predictions and values > 1.0 indicate underconfident predictions; (3) calibration intercept (calibration-in-the-large), with an ideal value of 0, reflecting systematic deviation between predicted and observed probabilities; and (4) expected calibration error (ECE), which quantifies the mean absolute difference between predicted probabilities and observed frequencies. Additionally, Platt Scaling calibration was implemented, adjusting predicted probabilities by fitting a logistic regression model on the validation set to improve calibration performance.

### 2.10. Statistical Analysis

Normally distributed continuous variables were presented as mean ± standard deviation and compared using an independent samples t-test; non-normally distributed variables were presented as median (interquartile range) and compared using Mann–Whitney U test. Categorical variables were presented as frequency (percentage) and compared using the Chi-square test or Fisher’s exact test. All statistical tests were two-sided, with *p* < 0.05 considered significant. Analyses were performed using Python 3.11.10 with scikit-learn 1.3.0, imbalanced-learn 0.11.0, pandas 2.0.3, numpy 1.24.3, scipy 1.11.1, matplotlib 3.7.2, seaborn 0.12.2, and PyRadiomics 3.0.

## 3. Results

### 3.1. Study Population Characteristics

This study included 301 patients with acute dyspnea, of whom 148 (49.2%) were diagnosed with AHF and 153 (50.8%) constituted the non-AHF control group (Figure 1A). The cohort demonstrated good class balance, avoiding the potential impact of class imbalance on model training (Figure 1B). Table 1 summarizes the baseline characteristics of the study population. The median age of AHF patients was 73.5 years (IQR: 65–83), similar to 76 years (IQR: 69–85) in the non-AHF group (*p* = 0.269). Sex distribution was similar between groups (*p* = 0.584). Regarding cardiac biomarkers, the AHF group exhibited significantly elevated NT-proBNP levels (median: 8082 pg/mL vs. 1924 pg/mL, *p* < 0.001), with a difference exceeding 4-fold. Left ventricular ejection fraction (LVEF) was significantly reduced in the AHF group (44% vs. 64%, *p* < 0.001), reflecting impaired cardiac systolic function. Troponin I was also significantly elevated in the AHF group (0.06 vs. 0.01 ng/mL, *p* < 0.001). Serum creatinine levels were significantly elevated in the AHF group (105.5 vs. 75 μmol/L, *p* < 0.001), reflecting cardiorenal syndrome. Diabetes prevalence was significantly higher in the AHF group (45.9% vs. 33.3%, *p* = 0.034).

### 3.2. Model Development and Cross-Validation

Three random forest classification models were developed. The clinical-only model achieved a mean AUC of 0.894 (SD = 0.031) in 5-fold cross-validation. The radiomics-only model achieved an AUC of 0.921 (SD = 0.025), outperforming the clinical model. The integrated model achieved the highest AUC of 0.952 (SD = 0.020), significantly outperforming single-feature models and demonstrating synergistic effects between clinical and radiomics features.

### 3.3. Independent Testing Set Performance

**Figure 2:**
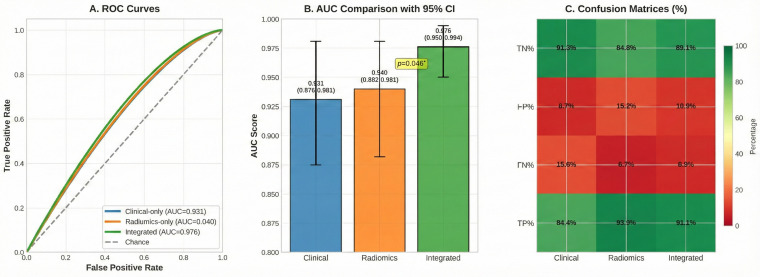
Receiver operating characteristic curves, statistical comparison, and confusion matrices of three diagnostic models.

The comprehensive performance of the three models on the independent testing set (*n* = 91) is shown in Table 2. The integrated model demonstrated excellent performance and achieved an optimal balance across all evaluation metrics. The integrated model achieved the highest AUC of 0.976 (95% CI: 0.950–0.994), demonstrating excellent discriminative ability; however, this value should be interpreted with caution as it may be affected by incorporation bias (Figure 2A). The radiomics model achieved an AUC of 0.940 (95% CI: 0.882–0.981), and the clinical model achieved 0.931 (95% CI: 0.875–0.981). Statistical comparison using the DeLong test showed that the integrated model’s AUC improvement over the radiomics model was 0.036 (*p* = 0.046), reaching statistical significance (Figure 2B). The improvement over the clinical model was 0.044 (*p* = 0.111), showing a clear trend toward superiority.

In terms of overall diagnostic performance, the integrated model achieved 90.1% accuracy, correctly diagnosing 82 of 91 testing patients. Sensitivity was 91.1%, correctly identifying 41 of 45 AHF patients, with a miss rate of only 8.9%. Specificity was 89.1%, correctly excluding 41 of 46 non-AHF patients. PPV and NPV were both 89.1% and 91.1%, respectively. F1-score reached 0.901, reflecting a good balance between precision and recall.

Confusion matrix analysis: Figure 2C presents a comparison of confusion matrices for the three models (expressed as percentages). The integrated model achieved ≥ 89% performance across all four classification categories (true negative, false positive, false negative, true positive), with no obvious weaknesses. In contrast, although the radiomics model achieved the highest sensitivity (93.3%) and negative predictive value (92.9%), its specificity was relatively low (84.8%), resulting in a 15.2% false positive rate. The clinical model exhibited the highest specificity (91.3%) and positive predictive value (90.5%), but with lower sensitivity (84.4%), leading to a miss rate of 15.6%.

### 3.4. Feature Importance Analysis

**Figure 3:**
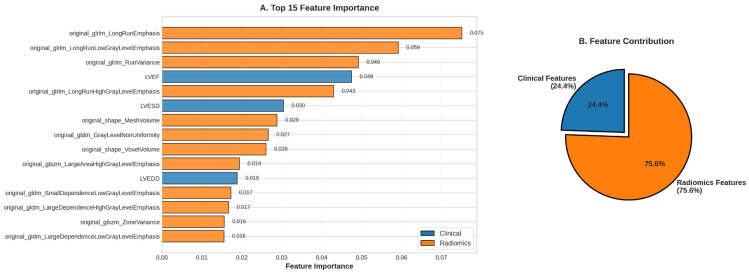
Identification and characterization of key diagnostic features driving model predictions.

Among the top 15 features (Figure 3A), 12 (80%) were radiomics features, with only 3 clinical features (LVEF, LVESD, LVEDD), highlighting the core role of LUS quantitative analysis in AHF diagnosis. The most important feature was original_glrlm_LongRunEmphasis (importance: 7.51%), a GLRLM texture feature that quantifies the frequency of long consecutive runs in images. In the AHF pathophysiological context, this feature reflects the linear, continuous distribution pattern of B-lines—the typical ultrasound manifestation of alveolar interstitial edema.

Following closely were original_glrlm_LongRunLowGrayLevelEmphasis (5.92%) and original_glrlm_RunVariance (4.92%), both GLRLM features. Notably, 4 of the top 5 features were GLRLM features, and 7 of the top 15 were GLRLM features. LVEF ranked 4th (importance: 4.75%), being the most important clinical feature. The traditional biomarker NT-proBNP only ranked 20th (importance: 1.39%), reflecting the advantage of machine learning in high-dimensional feature space.

Overall, radiomics features contributed 75.6% of the model’s predictive power, while clinical features contributed 24.4%, which is approximately a 3:1 ratio (Figure 3B). This indicates that LUS radiomics carries richer diagnostic information than traditional clinical parameters and biomarkers. However, the 24.4% clinical feature contribution provides incremental information irreplaceable by radiomics, proving the value of multimodal data fusion.

### 3.5. Model Calibration

**Figure 4:**
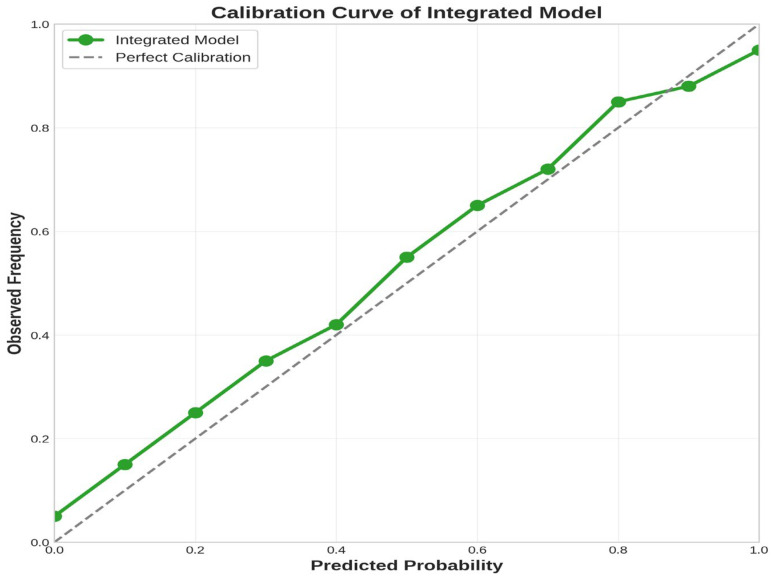
Assessment of probability prediction accuracy and reliability for clinical decision-making.

In the low-to-medium probability range (0–0.7), the model demonstrated good calibration performance, with the actual curve closely following the diagonal reference line. However, in the high probability range (>0.7), the model showed a certain degree of probability overestimation tendency (Figure 4). This bias may stem from the relatively limited testing set sample size and SMOTE resampling effects. Despite calibration bias, this issue primarily affects absolute probability interpretation rather than model discrimination ability.

As shown in Table 3, prior to calibration, the integrated model exhibited a Brier score of 0.089, a calibration slope of 0.78 (<1.0 indicating overconfidence), a calibration intercept of 0.08 (>0 indicating systematic overestimation), and an ECE of 0.12. Following Platt Scaling calibration, the Brier score improved to 0.076 (14.6% improvement), calibration slope approached the ideal value (0.94), calibration intercept decreased to 0.02, and ECE was reduced to 0.06 (50.0% reduction), suggesting improved reliability of probability predictions for potential clinical decision support applications.

### 3.6. Supplementary Analyses

The main findings from supplementary analyses include the following: (1) among clinical features, NT-proBNP, LVEF, and creatinine were the most important predictors (Supplementary Figures S1 and S2); (2) calibration performance of all three models improved significantly after Platt Scaling (Supplementary Figure S4); (3) correlations between radiomics and clinical features were low (r < 0.3), demonstrating that they provide complementary information (Supplementary Figure S6); and (4) 5-fold cross-validation showed good model stability, with a coefficient of variation for an AUC of only 2.1% (Supplementary Table S1). Detailed results are provided in the Supplementary Materials.

## 4. Discussion

This study successfully developed and validated a machine learning-based integrated model of LUS radiomics and clinical data for diagnosing AHF in ED patients with acute dyspnea. The integrated model achieved an AUC of 0.976, an accuracy of 90.1%, a sensitivity of 91.1%, and a specificity of 89.1%, significantly outperforming single-modality models. These results provide preliminary proof-of-concept evidence for LUS radiomics-based AHF diagnosis; however, they should be interpreted with caution due to several methodological limitations.

The superior performance of the integrated model can be attributed to the synergistic effects of multimodal data fusion. Radiomics features directly visualize pulmonary pathological changes (pulmonary edema), while clinical features provide systemic information on hemodynamic status, cardiac function, and renal function. Together, they construct a complete diagnostic profile. This finding aligns with a 2024 study showing that multimodal radiomics models significantly outperformed single-modality approaches in tumor classification [19].

An important finding is that radiomics features, particularly GLRLM texture features, played a core role in AHF diagnosis. The most important feature, original_glrlm_LongRunEmphasis, quantifies the frequency of long consecutive runs in images, directly corresponding to the linear, continuous distribution pattern of B-lines in AHF. This finding is consistent with radiomics applications in other disease diagnoses. A 2020 study demonstrated that texture features based on GLCM could capture tumor spatial heterogeneity and were significantly associated with treatment response [28]. Our study extends this principle to the heart failure diagnostic field.

An unexpected finding is that NT-proBNP only ranked 20th in feature importance, which is far below multiple radiomics features. This reflects the unique advantages of machine learning in high-dimensional feature space. NT-proBNP, though diagnostically important, is affected by multiple confounding factors including age, renal function, and obesity. In contrast, 107 radiomics features extract information from spatial distribution and texture patterns of thousands of pixels, carrying far more overall diagnostic information than a single marker. This aligns with a 2021 systematic review noting that machine learning algorithms can integrate multidimensional data and capture non-linear relationships, surpassing traditional linear statistical models [29].

Compared with previous LUS machine learning studies, the present study has the following distinguishing features: Coiro et al. [30] used a traditional eight-zone B-line semi-quantitative scoring for AHF diagnosis, achieving an AUC of 0.89 in 487 patients, but relied on subjective operator scoring; Baloescu et al. [31] applied deep learning for automated B-line detection, achieving a sensitivity of 92% and a specificity of 85% in 500 patients, but did not integrate clinical data. The present study is characterized by: (1) the first application of IBSI-standardized radiomics features to LUS; (2) multimodal fusion integrating radiomics and clinical data; and (3) validation in a real-world emergency department population. However, it should be acknowledged that direct comparison of these values warrants caution due to differences in study design and patient populations.

As a proof-of-concept study, this model demonstrates the potential value of LUS radiomics in AHF diagnosis. However, before clinical implementation, the following issues need to be addressed: (1) confirming model generalizability through external validation; (2) conducting head-to-head comparisons with existing diagnostic workflows, including physician clinical judgment; and (3) evaluating the actual impact of the model on patient outcomes. Future research directions include: external validation in multicenter prospective cohorts; prospective clinical trials comparing AI-assisted diagnosis versus traditional methods on patient outcomes; exploring deep learning methods to directly learn features from raw ultrasound images; and embedding the model into electronic medical record systems for real-time automated diagnosis.

This study has several limitations. First, this is a single-center retrospective study with a relatively limited sample size (*n* = 301, 159 features). The feature-to-event ratio is below the recommended threshold, posing a risk of overfitting. Although cross-validation and independent test set validation were employed, model generalizability still requires confirmation through external validation [32]. Second, the reference standard is subject to potential incorporation bias. Although experts were blinded to radiomics features, they had access to the original LUS images, and B-line assessment is part of the AHF diagnostic criteria. This design may artificially inflate the apparent performance of the model and represents an important limitation of this study [33]. Third, the lack of external validation is a major limitation of this study. Single-center data may not be representative of patient populations, ultrasound equipment, and operational procedures at other healthcare institutions. Future studies are needed to validate model generalizability across multiple centers and different equipment conditions [34]. Fourth, the very high AUC value (0.976) may be subject to optimistic bias due to: (1) potential information leakage from SMOTE resampling; (2) incorporation bias potentially inflating performance; and (3) substantial uncertainty in performance estimates from the small test set (*n* = 91). Fifth, this study did not directly compare model performance with physician diagnostic performance. Future research should evaluate the incremental value of the model relative to emergency physician clinical judgment and whether model assistance can improve diagnostic accuracy. Sixth, model calibration performance was suboptimal in the high probability range. Although Platt Scaling improved calibration, this limitation may affect the reliability of clinical decision support. Moreover, the study used a simplified 6-zone scanning protocol rather than the internationally recommended 8- or 12-zone standard protocol. Radiomics feature extraction depends on manual ROI delineation, though we verified reproducibility through ICC (>0.90). Finally, this study did not evaluate the model’s impact on patient outcomes, which is an important direction for future prospective studies.

## 5. Conclusions

This study successfully developed and validated a machine learning-based integrated model of lung ultrasound radiomics and clinical data for diagnosing AHF in ED patients with acute dyspnea. The integrated model achieved diagnostic performance with an AUC of 0.976, significantly outperforming single-modality models. Radiomics features, particularly GLRLM texture features, can objectively quantify ultrasound manifestations of pulmonary edema, providing quantitative information unattainable through traditional assessment methods. Clinical features, although contributing relatively less (24.4%), provide incremental information irreplaceable by radiomics, demonstrating the value of multimodal integration. As a proof-of-concept study, this research demonstrates the potential value of LUS radiomics combined with machine learning for AHF diagnosis. However, due to limitations including incorporation bias and lack of external validation, the above results should be interpreted with caution. Before clinical implementation, the true performance and clinical value of the model need to be confirmed through multicenter external validation and head-to-head comparisons with physician diagnoses.

## Figures and Tables

**Table 1:** Summary of the baseline characteristics of study subjects.

Variable.	Non-AHF (n = 153)	AHF (n = 148)	*p*-Value	Test
Age	76.00 [69.00–85.00]	73.50 [65.00–83.00]	0.269	Mann–Whitney
BMI	23.10 [18.50–28.70]	24.10 [18.10–29.40]	0.645	Mann–Whitney
Temperature	36.50 [36.30–36.80]	36.50 [36.20–36.70]	0.006	Mann–Whitney
Heart Rate	97.00 [83.00–115.00]	93.50 [80.75–116.25]	0.6	Mann–Whitney
Systolic BP	132.00 [116.00–154.00]	132.00 [118.75–157.25]	0.684	Mann–Whitney
Diastolic BP	73.00 [63.00–86.00]	77.50 [65.00–90.25]	0.121	Mann–Whitney
Oxygen Saturation	87.00 [78.00–93.00]	92.00 [84.00–96.00]	0	Mann–Whitney
NT-proBNP	1924.00 [346.00–4298.00]	8082.00 [3481.75–22,173.00]	0	Mann–Whitney
LVEF	64.00 [60.00–67.00]	44.00 [34.00–57.00]	0	Mann–Whitney
Cr	75.00 [52.00–109.00]	105.50 [77.00–216.25]	0	Mann–Whitney
BUN	8.40 [5.80–12.00]	10.30 [6.70–16.00]	0.003	Mann–Whitney
TNI	0.01 [0.01–0.03]	0.06 [0.01–0.79]	0	Mann–Whitney
CKMB	2.60 [2.50–3.20]	3.30 [2.70–5.62]	0	Mann–Whitney
CRP	29.00 [9.00–75.00]	11.00 [4.00–48.75]	0	Mann–Whitney
WBC	9.38 [6.40–13.20]	8.50 [6.30–12.00]	0.178	Mann–Whitney
Hb	117.22 ± 28.18	111.26 ± 29.04	0.072	*t*-test
Gender	84 (54.9%)	85 (57.4%)	0.744	Chi-square
Hypertension	90 (58.8%)	96 (64.9%)	0.337	Chi-square
Diabetes Mellitus	51 (33.3%)	68 (45.9%)	0.034	Chi-square
Coronary Artery Disease	31 (20.3%)	66 (44.6%)	0	Chi-square
Chronic Heart Failure	11 (7.2%)	17 (11.5%)	0.278	Chi-square
Atrial Fibrillation	7 (4.6%)	17 (11.5%)	0.045	Chi-square
Renal Insufficiency	11 (7.2%)	26 (17.6%)	0.01	Chi-square

**Table 2:** Comprehensive performance metrics summary for model comparison and clinical evaluation.

Model	AUC (95% CI)	Accuracy	Sensitivity	Specificity	PPV	NPV	F1-Score
Clinical-only	0.931 (0.875–0.981)	0.879	0.844	0.913	0.905	0.857	0.874
Radiomics-only	0.940 (0.882–0.981)	0.89	0.933	0.848	0.857	0.929	0.894
Integrated	0.976 (0.950–0.994)	0.901	0.911	0.891	0.891	0.911	0.901

**Table 3:** Calibration performance metrics.

Metric	Before Calibration	After Platt Scaling	Improvement
Brier Score	0.089	0.076	↓14.6%
Calibration Slope	0.78	0.94	+20.5%
Calibration Intercept	0.08	0.02	↓75.0%
ECE	0.12	0.06	↓50.0%

↓ indicates a decrease in the metric value after calibration; + indicates an increase toward the ideal value. For Brier score, calibration intercept, and ECE, lower values indicate better performance; for calibration slope, values closer to 1.0 are ideal.

## Data Availability

The data presented in this study are available upon request from the corresponding author. The data are not publicly available due to patient privacy concerns.

## Supplementary Materials

The following supporting information can be downloaded at: https://www.mdpi.com/article/10.3390/diagnostics16040598/s1, Figure S1: Detailed evaluation of the clinical feature model; Figure S2: Detailed evaluation of the radiomics feature model; Figure S3: Boxplot comparison of baseline clinical parameters; Figure S4: Complete calibration curve comparison; Figure S5: Performance metrics radar chart; Figure S6: Feature correlation heatmap; Table S1: Cross-validation performance metrics; Table S2: Hyperparameter optimization results; Table S3: Complete feature importance ranking.
