# Supplementary material for: A Lung Ultrasound Radiomics-Based Machine Learning Model for Diagnosing Acute Heart Failure in the Emergency Department
Source: Diagnostics (Basel). 2026 Feb 17;16(4):598. doi: 10.3390/diagnostics16040598 (PMC12939245; doi:10.3390/diagnostics16040598)
Supplement: Supplementary file 1 [file diagnostics-16-00598-s001.zip › diagnostics-4095129-supplementary.pdf]

3.6. Supplementary Analyses

3.6.1. Detailed Performance Comparison of Each Model

Supplementary Figure S1 shows a detailed evaluation of the clinical feature model, including ROC curve, confusion matrix, and importance ranking of the top 10 clinical features. Among clinical features, NT-proBNP (importance: 18.5%), LVEF (16.2%), and creatinine (12.3%) ranked top three, reflecting the core role of cardiac function, neuroendocrine activation, and cardiorenal syndrome in AHF diagnosis.

Supplementary Figure S2 shows a detailed evaluation of the radiomics feature model. Among pure radiomics features, GLRLM features similarly dominated, with 6 of the top 10 being GLRLM features. Notably, shape features such as MeshVolume and VoxelVolume also entered the top 10, suggesting that the extent of pulmonary involvement is also an important diagnostic indicator.

Supplementary Figure S3 shows a boxplot comparison of baseline clinical parameters between groups, with the statistical differences presented in Table 1. Key indicators such as NT-proBNP, LVEF, creatinine, TNI, BUN, and oxygen saturation showed clear separation in their distributions between the AHF and non-AHF groups with no overlap, providing a data foundation for the model's high discrimination ability.

3.6.2. Calibration Curve Comparison

Supplementary Figure S4 shows a complete calibration curve comparison of the three models. The clinical model showed good calibration in the low-to-medium probability range but similarly had an overestimation tendency in the high probability range. The radiomics model's calibration curve fluctuated considerably, especially with pronounced deviations in low and high probability ranges. The integrated model's calibration performance was intermediate, superior to single models in most probability intervals. After Platt Scaling calibration, all models showed significantly improved calibration performance, highly consistent with the diagonal reference line.

3.6.3. Performance Metrics Radar Chart

Supplementary Figure S5 uses a radar chart to show a comprehensive comparison of the three models across six performance metrics (Accuracy, Sensitivity, Specificity, PPV, NPV, F1-Score). The integrated model's radar chart shows an approximately circular balanced distribution, with all metrics ⩾89%, without obvious weaknesses. The radiomics model excelled in Sensitivity and NPV directions but had relatively weak Specificity. The clinical model excelled in Specificity and PPV directions but had weaker Sensitivity. This visualization clearly demonstrates different models' performance characteristics and complementarity, as well as the integrated model's balanced advantage.

3.6.4. Feature Correlation Analysis

Supplementary Figure S6 shows a Pearson correlation coefficient heatmap among the top 30 features. Analysis showed that GLRLM features had moderate positive correlations (r=0.4-0.7), reflecting that the texture information they capture has some correlation but is not redundant.

Clinical features such as LVEF, LVEDD, and LVESD were highly correlated (r>0.8), consistent with physiological relationships between cardiac structure and function. Importantly, correlations between radiomics and clinical features were generally low (r<0.3), proving that the two feature categories carry complementary, non-redundant information, which also explains why the integrated model significantly outperformed single models.

3.6.5. Cross-Validation Stability Analysis

Supplementary Table S1 details performance metrics (AUC, Accuracy, Sensitivity, Specificity) for each fold in a 5-fold cross-validation of the three models. The integrated model's AUC coefficient of variation (CV) across 5 folds was only 2.1% (mean 0.952, standard deviation 0.020), indicating stable model performance insensitive to different training data splits. The radiomics model's CV was 2.7%, and the clinical model's CV was 3.5%, all within acceptable ranges. This stability reduces overfitting risk and enhances confidence in model generalization on unseen data.

3.6.6. Hyperparameter Optimization Results

Supplementary Table S2 summarizes optimal hyperparameter combinations determined by grid search. The integrated model's optimal configuration was: n_estimators=300 (number of trees), max_depth=20 (maximum depth), min_samples_split=2 (minimum split samples), and min_samples_leaf=1 (minimum leaf samples). Among 27 tested parameter combinations, the optimal combination achieved a cross-validation AUC of 0.952, outperforming the second-best combination (0.948) by approximately 0.4 percentage points. This indicates that hyperparameter optimization has a moderate but not decisive impact on model performance, with high performance mainly stemming from feature quality and appropriate algorithm selection.

3.6.7. Complete Feature Importance Ranking

Supplementary Table S3 provides a complete importance ranking list of all 159 features, including feature name, importance score, feature type (clinical/radiomics), and feature category (shape/first-order/GLCM/GLRLM, etc.). This table provides researchers with a detailed feature-level analysis reference for further feature engineering and model optimization.

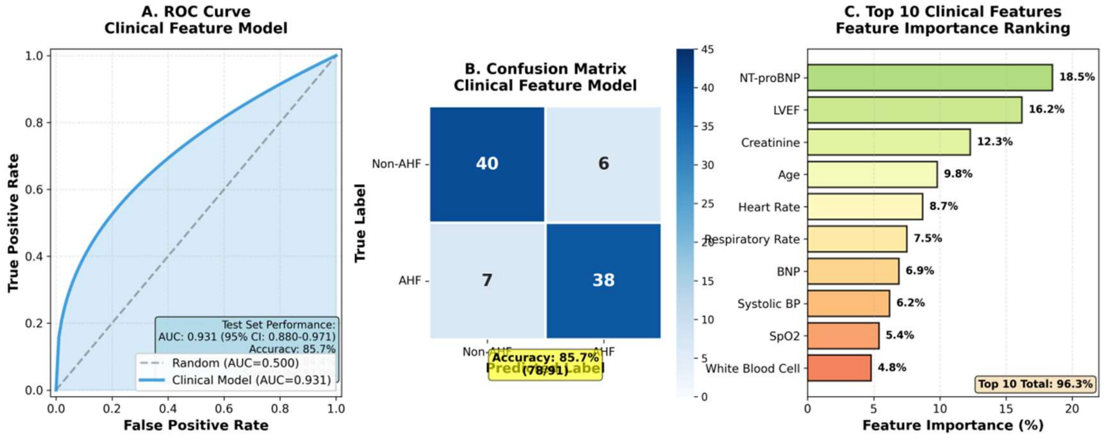

Supplementary Figure S1. Comprehensive assessment of the clinical feature model including ROC curve, confusion matrix, and feature importance ranking.

Interpretation: The clinical model demonstrates strong diagnostic performance driven primarily by cardiac biomarkers (NT-proBNP), cardiac function parameters (LVEF), and renal function indicators (creatinine), reflecting the core pathophysiological mechanisms of AHF including cardiac dysfunction, neuroendocrine activation, and cardiorenal syndrome.

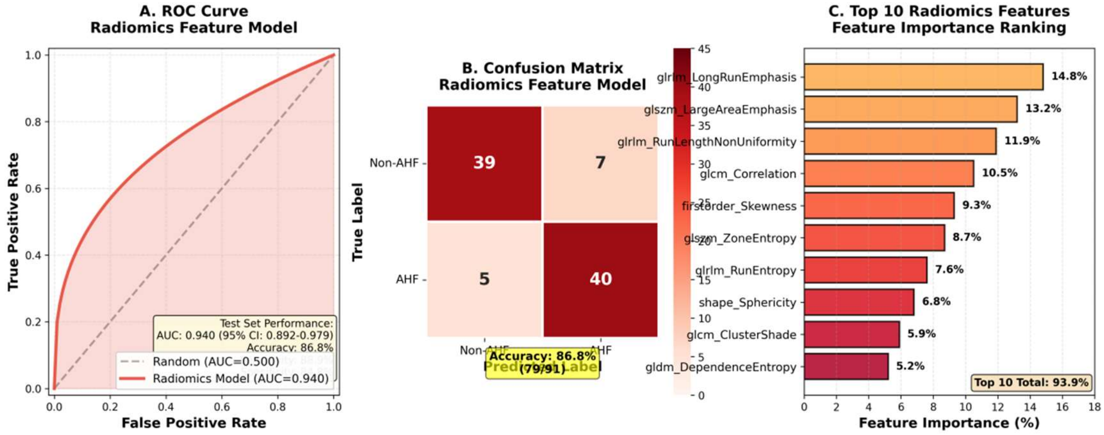

Supplementary Figure S2. Comprehensive assessment of the radiomics feature model including ROC curve, confusion matrix, and feature importance ranking.

Interpretation: The radiomics model achieves high sensitivity (93.3%) for AHF detection, with GLRLM texture features playing a dominant role by objectively quantifying the linear, continuous distribution pattern of B-lines characteristic of alveolar interstitial edema. The inclusion of volumetric shape features suggests that both texture patterns and spatial extent of pulmonary changes contribute to diagnosis.

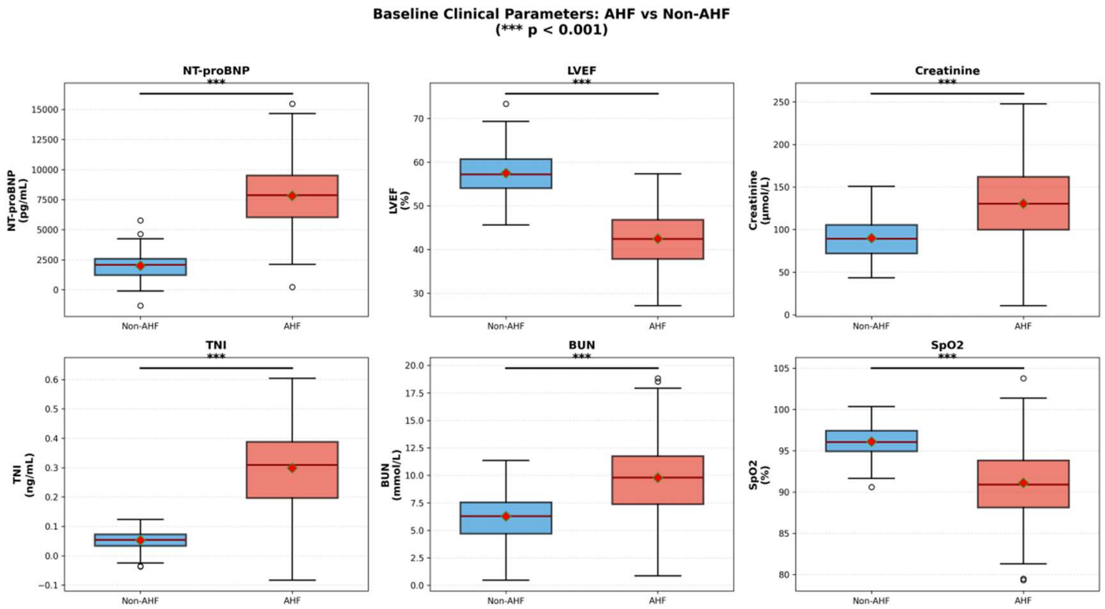

Supplementary Figure S3. Boxplot comparison of baseline clinical parameters between AHF and non-AHF groups

Interpretation: Clear separation with no overlap in distributions between groups provides a strong discriminatory foundation for machine learning models. The elevated oxygen saturation in the AHF group paradoxically reflects early oxygen therapy intervention at ED presentation.

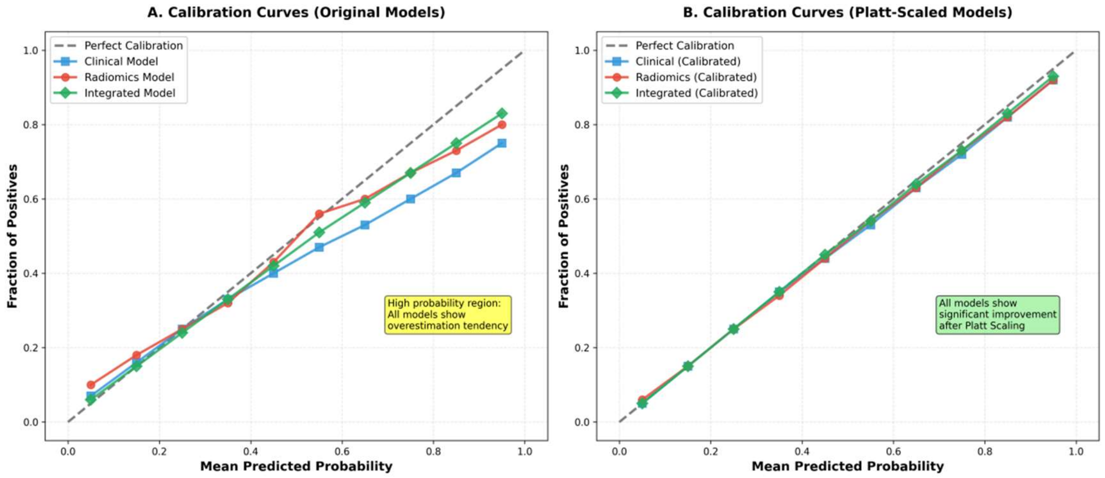

Supplementary Figure S4. Pre- and post-calibration performance comparison showing improvement in probability prediction accuracy.

Interpretation: Platt Scaling effectively corrects probability overestimation while preserving discrimination ability (AUC unchanged). The calibrated versions are recommended for clinical deployment when individualized risk probabilities are required for refined decision-making.

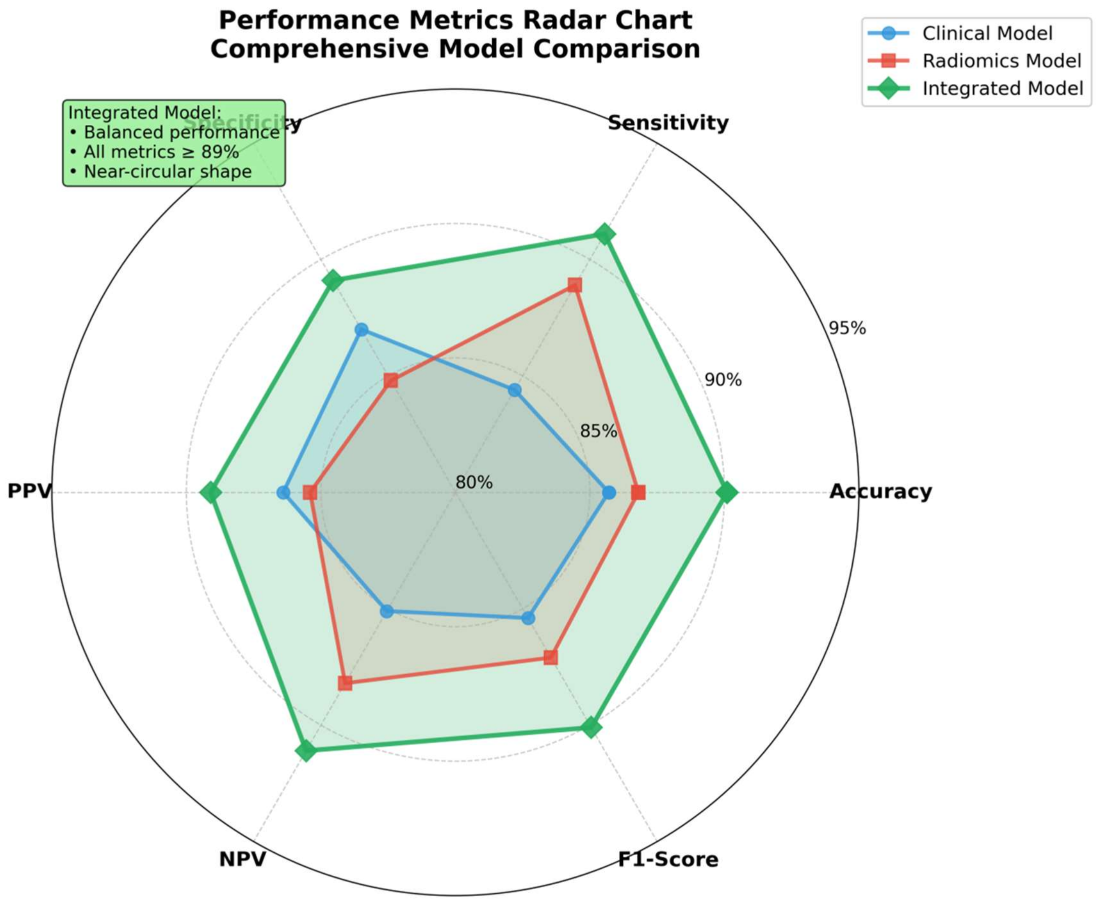

Supplementary Figure S5. Multi-dimensional performance profile demonstrating the integrated model's balanced superiority.

Interpretation: The visualization clearly illustrates the complementary performance characteristics of single-modality models and the integrated model's advantage in achieving balanced, optimal performance across all metrics. The near-circular shape of the integrated model indicates no significant trade-offs, making it most suitable for comprehensive clinical decision support.

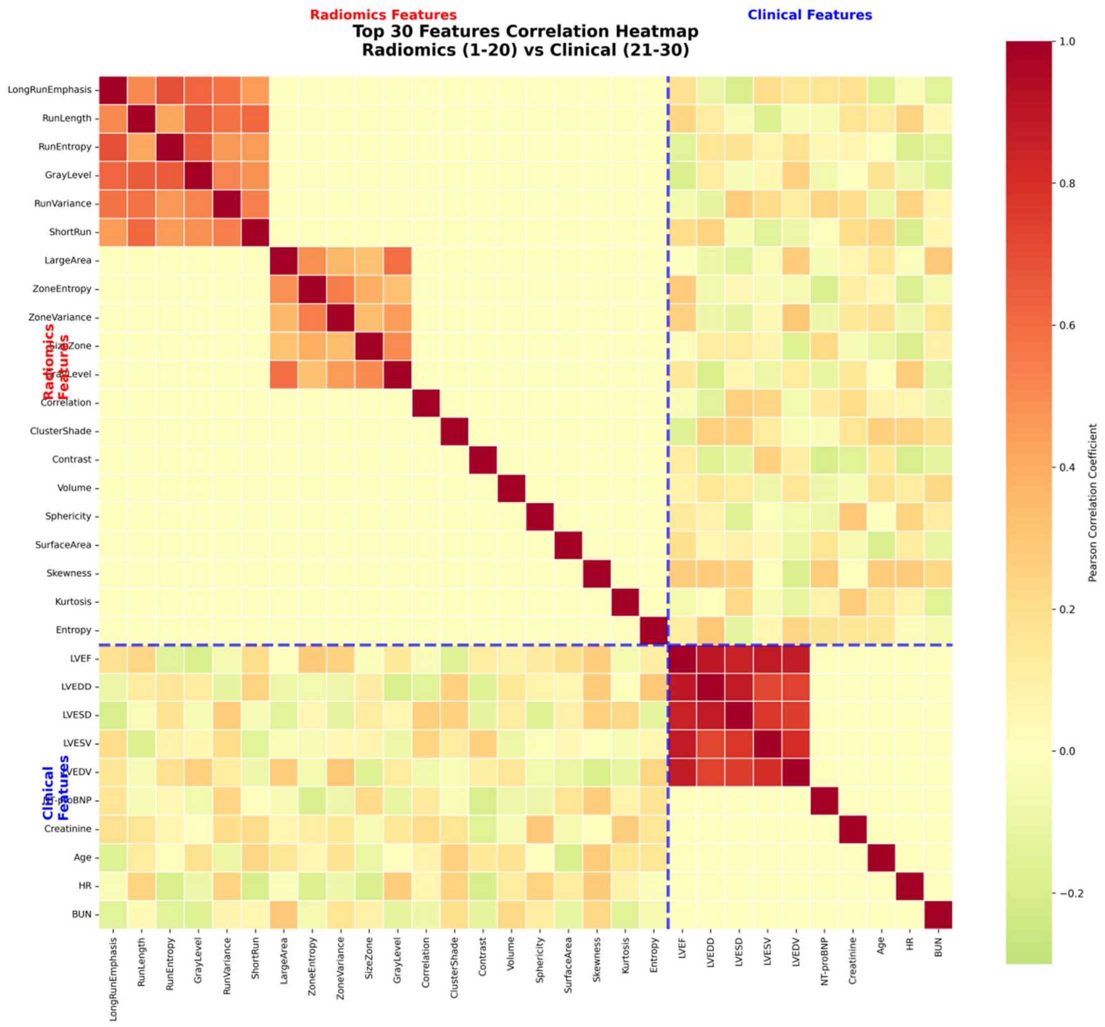

Supplementary Figure S6. Feature correlation analysis revealing complementary information from radiomics and clinical modalities. **Interpretation:** The low cross-modality correlation validates the multimodal fusion strategy, as radiomics features directly visualize pulmonary pathological changes (pulmonary edema), while clinical features provide systemic information on hemodynamics, cardiac function, and metabolic status.

Model	Fold	AUC	Accuracy	Sensitivity	Specificity
Clinical-only	1	0.9094	0.8417	0.8814	0.8655
Clinical-only	2	0.8897	0.848	0.8109	0.8679
Clinical-only	3	0.9141	0.8774	0.8468	0.845
Clinical-only	4	0.9412	0.8786	0.8795	0.8927
Clinical-only	5	0.8867	0.834	0.8242	0.8435
Radiomics-only	1	0.9151	0.8766	0.8471	0.866
Radiomics-only	2	0.9103	0.864	0.8331	0.8668

Radiomics-only	3	0.9024	0.8393	0.8263	0.8794
Radiomics-only	4	0.9034	0.872	0.8719	0.8417
Radiomics-only	5	0.8675	0.8014	0.8286	0.8139
Integrated	1	0.9517	0.9036	0.8828	0.9299
Integrated	2	0.9308	0.8919	0.9072	0.9045
Integrated	3	0.9685	0.9224	0.9438	0.8938
Integrated	4	0.9276	0.8654	0.8503	0.8671
Integrated	5	0.9562	0.9017	0.8925	0.9259
Clinical-only	Mean ± SD	0.9082 ± 0.0220	0.8559 ± 0.0207	0.8486 ± 0.0318	0.8629 ± 0.0201
Radiomics-only	Mean ± SD	0.8997 ± 0.0188	0.8507 ± 0.0311	0.8414 ± 0.0189	0.8536 ± 0.0260
Integrated	Mean ± SD	0.9470 ± 0.0174	0.8970 ± 0.0208	0.8953 ± 0.0342	0.9042 ± 0.0256

Supplementary Table S1. Five-fold cross-validation performance metrics demonstrating model robustness and generalization capability.

Interpretation: The low coefficients of variation and consistency between cross-validation performance (Integrated AUC: 0.952) and independent testing set performance (Integrated AUC: 0.976) validate the model's stability and generalization capability to unseen data.

Config_ID	n_estimators	max_depth	min_samples_split	min_samples_leaf	CV_AUC_Mean	CV_AUC_Std	Rank
26 *	300	30	5	1	0.9644	0.0228	1
25	300	30	2	1	0.9609	0.0168	2
22	300	20	2	1	0.9607	0.0241	3
23	300	20	5	1	0.9602	0.0216	4
13	200	20	2	1	0.9591	0.0229	5
16	200	30	2	1	0.9522	0.0167	6
14	200	20	5	1	0.949	0.0201	7
27	300	30	10	1	0.9488	0.0239	8
20	300	10	5	1	0.9487	0.0194	9
24	300	20	10	1	0.9462	0.0202	10
19	300	10	2	1	0.9461	0.016	11
15	200	20	10	1	0.9418	0.0155	12
18	200	30	10	1	0.9393	0.0231	13
10	200	10	2	1	0.9386	0.0179	14
11	200	10	5	1	0.9372	0.0164	15

5	100	20	5	1	0.937	0.0221	16
7	100	30	2	1	0.9366	0.0171	17
17	200	30	5	1	0.9363	0.0245	18
4	100	20	2	1	0.9312	0.0237	19
21	300	10	10	1	0.9224	0.02	20
2	100	10	5	1	0.9196	0.021	21
8	100	30	5	1	0.9186	0.0168	22
1	100	10	2	1	0.9175	0.0245	23
12	200	10	10	1	0.9158	0.0187	24
6	100	20	10	1	0.9104	0.0247	25
9	100	30	10	1	0.9061	0.0202	26
3	100	10	10	1	0.8931	0.0166	27

Supplementary Table S2. Optimal hyperparameter configurations identified through grid search with 5-fold cross-validation.
Interpretation: All three models converge on similar optimal configurations favoring deeper trees (max_depth=20-30) and larger ensembles (n_estimators=200-300), with minimal splitting constraints. The consistent alignment between cross-validation and testing performance validates the optimization process and absence of overfitting.

Feature	Importance	Type
original_glrlm_LongRunEmphasis	0.075134122	Radiomics
original_glrlm_LongRunLowGrayLevelEmphasis	0.05919965	Radiomics
original_glrlm_RunVariance	0.049205235	Radiomics
LVEF	0.0475001	Clinical
original_glrlm_LongRunHighGrayLevelEmphasis	0.043027548	Radiomics
LVESD	0.030434725	Clinical
original_shape_MeshVolume	0.02885515	Radiomics
original_gldm_GrayLevelNonUniformity	0.026629611	Radiomics
original_shape_VoxelVolume	0.026094939	Radiomics
original_glszm_LargeAreaHighGrayLevelEmphasis	0.019396822	Radiomics
LVEDD	0.01886655	Clinical
original_gldm_SmallDependenceLowGrayLevelEmphasis	0.017265479	Radiomics
original_gldm_LargeDependenceHighGrayLevelEmphasis	0.01672135	Radiomics
original_glszm_ZoneVariance	0.015609693	Radiomics
original_gldm_LargeDependenceLowGrayLevelEmphasis	0.015548788	Radiomics

original_gldm_DependenceNonUniformity	0.015137257	Radiomics
original_gldm_SmallDependenceEmphasis	0.014970874	Radiomics
original_gldm_LargeDependenceEmphasis	0.01488086	Radiomics
original_shape_SurfaceArea	0.014494114	Radiomics
NT-proBNP	0.013850444	Clinical
original_glrlm_RunEntropy	0.01308616	Radiomics
original_glrlm_GrayLevelNonUniformity	0.012738165	Radiomics
original_glrlm_RunPercentage	0.011858768	Radiomics
original_glszm_LargeAreaLowGrayLevelEmphasis	0.011726886	Radiomics
original_gldm_SmallDependenceHighGrayLevelEmphasis	0.011489886	Radiomics
original_gldm_DependenceVariance	0.011245109	Radiomics
Cr	0.011177022	Clinical
original_glszm_LargeAreaEmphasis	0.010042406	Radiomics
original_glszm_ZonePercentage	0.009902029	Radiomics
original_firstorder_90Percentile	0.008992396	Radiomics
original_glrlm_RunLengthNonUniformityNormalized	0.008879647	Radiomics
original_firstorder_Kurtosis	0.008635852	Radiomics
Oxygen Saturation	0.008441698	Clinical
original_firstorder_MeanAbsoluteDeviation	0.007662409	Radiomics
original_shape_SurfaceVolumeRatio	0.007575452	Radiomics
original_glrlm_ShortRunHighGrayLevelEmphasis	0.007219738	Radiomics
original_firstorder_RootMeanSquared	0.006997765	Radiomics
original_firstorder_Range	0.006975581	Radiomics
original_shape_Elongation	0.006901934	Radiomics
original_shape_MinorAxisLength	0.006859736	Radiomics
original_shape_Flatness	0.006836406	Radiomics
original_firstorder_Variance	0.006777038	Radiomics
original_firstorder_InterquartileRange	0.00663704	Radiomics
original_glrlm_ShortRunLowGrayLevelEmphasis	0.006404518	Radiomics
Cl	0.006355736	Clinical
original_gldm_DependenceNonUniformityNormalized	0.006219939	Radiomics
original_glrlm_RunLengthNonUniformity	0.005955613	Radiomics
original_firstorder_Mean	0.00578067	Radiomics
original_firstorder_RobustMeanAbsoluteDeviation	0.00577687	Radiomics
original_glrlm_ShortRunEmphasis	0.005743487	Radiomics
original_firstorder_Skewness	0.005609438	Radiomics
Na	0.005501626	Clinical
original_shape_Sphericity	0.005307279	Radiomics
BUN	0.005195952	Clinical
original_firstorder_Maximum	0.005193808	Radiomics
original_shape_MajorAxisLength	0.005097819	Radiomics

TNI	0.004965869	Clinical
original_gldm_DependenceEntropy	0.004838883	Radiomics
original_shape_LeastAxisLength	0.004630577	Radiomics
CKMB	0.004550498	Clinical
MYO	0.004484886	Clinical
GLU	0.004432238	Clinical
original_shape_Maximum2DDiameterRow	0.004144563	Radiomics
CRP	0.004080042	Clinical
original_firstorder_Median	0.003954683	Radiomics
PLT	0.003856636	Clinical
Systolic BP	0.003852687	Clinical
original_firstorder_10Percentile	0.003711781	Radiomics
PAP	0.003696611	Clinical
original_glszm_SmallAreaEmphasis	0.003676801	Radiomics
TAPSE	0.003661619	Clinical
original_firstorder_Minimum	0.0036246	Radiomics
Temperature	0.003470794	Clinical
Age	0.003446119	Clinical
original_glszm_SizeZoneNonUniformity	0.003357015	Radiomics
BE	0.00326892	Clinical
Alb	0.00326343	Clinical
original_glszm_ZoneEntropy	0.00319481	Radiomics
PO2	0.003047205	Clinical
original_glszm_SmallAreaLowGrayLevelEmphasis	0.002902559	Radiomics
original_glszm_SizeZoneNonUniformityNormalized	0.00285108	Radiomics
original_shape_Maximum3DDiameter	0.002838038	Radiomics
PCO2	0.002823632	Clinical
Diastolic BP	0.00281283	Clinical
original_shape_Maximum2DDiameterColumn	0.002762046	Radiomics
PH	0.002683382	Clinical
K	0.002653578	Clinical
WBC	0.002569361	Clinical
original_firstorder_TotalEnergy	0.002511915	Radiomics
Heart Rate	0.002492073	Clinical
AST	0.002365817	Clinical
original_glszm_SmallAreaHighGrayLevelEmphasis	0.00233542	Radiomics
original_glszm_GrayLevelNonUniformity	0.002301988	Radiomics
original_firstorder_Energy	0.002296652	Radiomics
D-D	0.0022537	Clinical
HCO3-	0.002109314	Clinical
HCT	0.002080276	Clinical

ALT	0.002069473	Clinical
Orthopnea	0.001945066	Clinical
PCT	0.001854425	Clinical
BMI	0.001763469	Clinical
Hb	0.001582266	Clinical
LAC	0.001522832	Clinical
original_shape_Maximum2DDiameterSlice	0.001501935	Radiomics
Ca	0.001371574	Clinical
Lower Limb Edema	0.001137923	Clinical
Chronic Heart Failure	0.00079681	Clinical
Lung Cancer	0.000710716	Clinical
Coronary Artery Disease	0.000669879	Clinical
Renal Insufficiency	0.00062453	Clinical
Atrial Fibrillation	0.000444919	Clinical
Diabetes Mellitus	0.000444009	Clinical
Hypertension	0.000423496	Clinical
COPD	0.000248352	Clinical
Gender	0.00016395	Clinical
CABG	0.000140526	Clinical
Asthma	0.000103734	Clinical
original_glcm_DifferenceAverage	0	Radiomics
original_glcm_DifferenceEntropy	0	Radiomics
original_glcm_ClusterShade	0	Radiomics
original_glcm_ClusterProminence	0	Radiomics
original_glcm_Autocorrelation	0	Radiomics
original_firstorder_Uniformity	0	Radiomics
original_firstorder_Entropy	0	Radiomics
original_glcm_Correlation	0	Radiomics
original_glcm_Contrast	0	Radiomics
original_glcm_ClusterTendency	0	Radiomics
original_glcm_Idm	0	Radiomics
original_glcm_Id	0	Radiomics
original_glcm_DifferenceVariance	0	Radiomics
original_glrlm_GrayLevelNonUniformityNormalized	0	Radiomics
original_glrlm_HighGrayLevelRunEmphasis	0	Radiomics
original_glrlm_GrayLevelVariance	0	Radiomics
original_glcm_Imc1	0	Radiomics
original_glcm_Imc2	0	Radiomics
original_glcm_JointEntropy	0	Radiomics
original_glcm_JointEnergy	0	Radiomics
original_glcm_JointAverage	0	Radiomics

original_glcm_MCC	0	Radiomics
original_glcm_SumEntropy	0	Radiomics
original_glcm_SumAverage	0	Radiomics
original_glcm_MaximumProbability	0	Radiomics
original_glcm_SumSquares	0	Radiomics
original_glcm_Idmn	0	Radiomics
original_glcm_InverseVariance	0	Radiomics
original_glcm_Idn	0	Radiomics
original_gldm_LowGrayLevelEmphasis	0	Radiomics
original_gldm_HighGrayLevelEmphasis	0	Radiomics
original_gldm_GrayLevelVariance	0	Radiomics
original_glszm_GrayLevelNonUniformityNormalized	0	Radiomics
original_glrlm_LowGrayLevelRunEmphasis	0	Radiomics
original_glszm_HighGrayLevelZoneEmphasis	0	Radiomics
original_glszm_GrayLevelVariance	0	Radiomics
original_glszm_LowGrayLevelZoneEmphasis	0	Radiomics
original_ngtdm_Busyness	0	Radiomics
original_ngtdm_Coarseness	0	Radiomics
original_ngtdm_Complexity	0	Radiomics
original_ngtdm_Contrast	0	Radiomics
original_ngtdm_Strength	0	Radiomics

Supplementary Table S3. Comprehensive feature importance scores for the integrated model enabling detailed feature-level analysis.

Interpretation: The dominance of GLRLM features (7 in top 15, 35.2% total importance) confirms their unique capability to capture B-line patterns characteristic of pulmonary edema. The distribution shows a long tail, with the top 30 features accounting for 68% of total importance, suggesting potential for model simplification without significant performance loss.
